# Platelet aggregation responses to *Salmonella* Typhimurium are determined by host anti-*Salmonella* antibody levels

**DOI:** 10.1080/09537104.2024.2437241

**Published:** 2024-12-16

**Authors:** Rachel E Lamerton, Samantha J Montague, Marisol Perez-Toledo, Steve P Watson, Adam F Cunningham

**Affiliations:** 1 Department of Cardiovascular Science, College of Medicine and Health, University of Birmingham, Birmingham, UK; 2 Department of Immunology and Immunotherapy, College of Medicine and Health, University of Birmingham, Birmingham, UK

**Keywords:** Antibodies, host–pathogen interactions, platelets, *Salmonella*, thromboinflammation

## Abstract

Invasive non-typhoidal *Salmonella* infections are responsible for >75 000 deaths/year and >500 000 cases/year globally. Seventy-five percent of these cases occur in Sub-Saharan Africa, an increasing number of which are from multi-drug resistant strains. Interactions between bacteria and platelets can lead to thrombus formation, which can be beneficial for control of infection (immunothrombosis), or harmful through uncontrolled inflammation and organ damage (thromboinflammation). It is unknown whether *Salmonella* Typhimurium can activate human platelets. To assess this, light transmission aggregometry was used to measure platelet activation by two different *Salmonella* Typhimurium strains in 26 healthy donors in platelet-rich plasma and washed platelets. In platelet-rich plasma, but not in washed platelets, *Salmonella* Typhimurium activated platelets in a donor- and strain-dependent manner mediated through the low affinity immune receptor FcγRIIA and the feedback agonists, ADP and thromboxane A_2_. Plasma swap studies between strong and weak responders demonstrated a plasma component was responsible for the variation between donors. Depletion of anti-*Salmonella* antibodies from plasma abolished *Salmonella-*induced platelet aggregation responses, and addition of polyclonal anti-*Salmonella* antibody allowed aggregation in washed platelets. Correlating levels of anti-*Salmonella* total IgG or the IgG1, IgG2, IgG3 and IgG4 subclasses to platelet responses revealed total IgG levels, rather than levels of individual subclasses, positively correlated with maximum platelet aggregation results, and negatively with lag times. Overall, we show that anti-*Salmonella* IgG antibodies are responsible for donor variation in platelet aggregation responses to *Salmonella* and mediate this activity through FcγRIIA.

## Introduction

As well as their role in hemostasis and thrombosis, platelets play an important role in immunity, with the ability to influence the immune response in multiple ways, from secretion of immunomodulatory molecules to interactions with leukocytes.^1^ Furthermore, the formation of platelet-containing clots can help trap bacteria, inhibiting their dissemination, in a process termed immunothrombosis.^2^ However, these clots can also influence the local inflammatory environment, and dysregulation of this can lead to a thromboinflammatory response in which thrombus formation becomes pathological.^3^ Therefore, platelet activation can result in beneficial or harmful outcomes for the host.

Invasive non-typhoidal *Salmonella* (iNTS) infections are a major cause of bacteremia in sub-Saharan Africa.^4,5^ Patients typically present with fever, with a range of other diverse symptoms possible, from splenomegaly to severe anemia.^6^ The highest incidence of infection occurs in the under 5s, with the immunocompromised and the elderly also disproportionately affected.^6,7^ In contrast to the non-typhoidal *Salmonella* serovars present across the world that mainly cause self-limiting gastroenteritis in immunocompetent individuals, iNTS bacteria are able to cross the gut barrier and enter the bloodstream. These strains are now the dominant isolates in many sub-Saharan regions and are becoming increasingly multi-drug resistant.^8^ *Salmonella enteritidis* serovar Typhimurium (hereon written as *Salmonella* Typhimurium, STm) accounts for over 77% of iNTS isolates serotyped in Africa^7^ and the representative epidemic STm strain D23580 is resistant to ampicillin, chloramphenicol, streptomycin, trimethoprim and sulfonamide, making treatment of this life-threatening infection problematic. The Global Burden of Disease Study estimated there were 535 000 cases of iNTS worldwide in 2017, leading to over 77 500 deaths,^9^ with many case reports describing thrombotic events concurrent with *Salmonella* infections of various serovars.^10–15^

Previous studies assessing the molecular interactions between bacteria and platelets have identified platelet receptors GPIbα, αIIbβ3, TLR2 and FcγRIIA as being involved in binding to certain bacterial strains.^16–19^ However, whilst there have been many studies looking at human platelet interactions with Gram-positive bacterial strains, there are far fewer studies investigating Gram-negative bacterial strains, with little research into non-typhoidal *Salmonella* strains. The only studies have looked at the effect of *Salmonella* Typhimurium on ADP-induced platelet aggregation, with one finding that purified filtered STm could inhibit ADP-induced aggregation (strain dependent),^20^ and the other finding that purified STm porins could enhance both ADP and thrombin-induced platelet aggregation.^21^

The aim of this study therefore was to assess whether two different strains of STm could activate human platelets.

## Methods

### Preparation of bacteria

One colony of *Salmonella* Typhimurium SL1344 (a standard laboratory strain^22^) or D23580 (an invasive African isolate^23,24^) was grown up overnight in 10 ml LB broth at 37°C. Cultures were then diluted 1:5 in LB broth and incubated for 2 h in an orbital shaking incubator at 37°C, 200 rpm, to obtain log-phase bacteria. Bacteria were spun at 10 000 *g* for 5 min, pellets resuspended in PBS, then centrifuged and resuspended twice more to wash bacteria. At the final resuspension, PBS was added to give a final OD_600_ of 1, relating to approximately 1 × 10^9^ CFU/ml bacteria.

### Blood collection and ethics

Blood was taken from healthy consenting volunteers under ethical approval ERN_11–0175 granted by the University of Birmingham Internal Ethical Review Panel and in accordance with the Declaration of Helsinki.

Venous blood (from donors who had not taken aspirin or non-steroidal anti-inflammatory drugs in the previous 10 days) was collected into 3.2% or 3.8% sodium citrate vacuette containers using a 21 gauge needle.

### Preparation of platelets

#### Platelet rich and platelet poor plasma

Blood taken into 3.2% sodium citrate was centrifuged at room temperature for 20 min at 200 *g*. The platelet-rich plasma (PRP) supernatant was removed before centrifuging the remaining buffy coat layer and red cells for 10 min at 1000 *g* to collect platelet poor plasma (PPP).

#### Washed platelets

Blood taken into 3.8% sodium citrate was further anticoagulated with 10% acid citrate dextrose (ACD, 120 mM sodium citrate, 110 mM glucose, 80 mM citrate dextrose, pH 4.4) before centrifugation for 20 min at 200 *g*. The PRP supernatant was collected and 0.2 μg/ml prostacyclin added before further centrifugation for 10 min at 1000 g. After discarding the supernatant, the pellet was resuspended in modified Tyrode’s (134 mM NaCl, 0.34 mM Na_2_HPO_4_.12 h_2_O, 2.9 mM KCl, 12 mM NaHCO_3_, 20 mM HEPES, 5 mM glucose, 1 mM MgCl_2_; pH 7.4) plus 3 ml ACD and 0.2 μg/ml prostacyclin and centrifuged for 10 min at 1000 g. The supernatant was discarded and platelet pellet resuspended in modified Tyrode’s to a concentration of 2 × 10^8^ platelets/ml. Platelets were rested for 30 min prior to experiments.

#### ADP sensitive washed platelets

Blood taken into 3.2% sodium citrate was further anticoagulated with 22% ACD and centrifuged for 20 min at 200 *g*. Supernatant (PRP) was collected and a further 10% ACD was added before centrifugation for 15 min at 500 *g*. The supernatant was removed, and the surface of the platelet pellet washed gently 3 times with Krebs-RingercGlucose buffer (120 mM NaCl, 4.9 mM KCl, 1.2 mM MgSO_4_, 1.7 mM KH_2_PO_4_, 8.3 mM Na_2_HPO_4_, 10 mM glucose, pH 7.4) supplemented with 0.01 U/ml grade 7 apyrase. For washed platelet assays, the pellet was resuspended in the buffer used to wash to a concentration of 2 × 10^8^ platelets/ml and 1 mM calcium chloride was added. For donor swap and antibody depletion assays, pellets were resuspended in ACD-free PPP supplemented withc 0.01 U/ml grade 7 apyrase (antibody depleted where stated), and adjusted to 2–3 × 10^8^ platelets/ml.

Platelet counts were measured using either a Coulter Counter or XP-300 (Sysmex).

### Antibody depletion of PPP

PPP was obtained as above, aliquoted and frozen at −20°C for up to 2 weeks. STm SL1344 was grown as above and normalized to OD_600_ = 1. Bacteria and thawed PPP were combined at a 1:1 volume ratio and rotated gently for 2 h at 4°C. Centrifugation for 5 min at 10 000 *g* was carried out to remove bacteria (and associated antibodies), with the resulting SL1344-binding anti-body depleted (dPPP) supernatant stored overnight at 4°C before use the following day. The ADP sensitive platelet preparation above was used to wash platelets, before resuspending pellet in either thawed PPP or dPPP.

### Platelet aggregometry

A PAP-8 aggregometer (Bio-data Corp) was used for all experiments, with 1200 rpm stirring and 37°C maintained throughout. Traces were recorded for at least 30 min. Donor-specific PPP was used as a blank for PRP experiments, or ADP sensitive platelet assays resuspended in PPP. Tyrode’s buffer was used as a blank for washed platelet assays. Where indicated, platelets were incubated with inhibitor for at least 3 min prior to start of recording.

### Anti-Salmonella antibody ELISA

To detect antibodies to *Salmonella* Typhimurium, bacteria were cultured as above and the pellets resuspended in PBS with 0.01% azide. The protein concentrations of washed bacteria were quantified using a Pierce™ BCA protein assay kit. 1 μg *Salmonella* in PBS was added per well to Nunc MaxiSorp 96 well plates and incubated at 4°C overnight. All subsequent steps were carried out at room temperature. Plates were washed 3 times with PBS before blocking with 2% (w/v) BSA in PBS-0.1% Tween 20 for 1 h. Plates were washed with PBS-0.1% Tween 20, three times, with this wash being carried out between all subsequent steps. Serum was diluted 1 in 20 in 2% (w/v) BSA in PBS-0.1% Tween 20, and incubated on plate for 1 h. 100 μl of the appropriate mouse anti-human HRP-conjugated secondary antibody diluted 1:4000 (unless stated) was added and plates incubated for 1 h: Total IgG (clone 2040–05; 1:8000), IgG1-Fc (clone HP6001), IgG2-Fc (clone 31-7-4), IgG3-hinge (clone HP6050) and IgG4-Fc (clone HP6025), all from Southern Biotech. Plates were developed with 100 μl TMB-Core (Bio-Rad) for up to 10 min, and the reaction stopped with 50 μl 0.2 M H_2_SO_4_. OD_450_ was read using a SpectraMax ABS Plus plate reader.

### Statistics

Statistical analysis was performed using Graph Pad Prism 9.4.1. Results are presented as stated in figure legends, either as mean ± SD or median ± interquartile range (IQR). Significance assumed at *p* < .05. Shapiro-Wilk was used to assess normality of data. To test for differences between two groups, paired t-tests and Wilcoxon tests were carried out for parametric and non-parametric data respectively. To test for correlations, Pearsons correlation coefficient or Spearman rank correlation were calculated for parametric and non-parametric data respectively.

## Results

### *Salmonella* can activate platelets in platelet rich plasma in a donor- and strain-dependent manner

In this study, we have used two well-characterized strains of STm. SL1344 is a standard laboratory, virulent, noninvasive strain isolated from cattle,^22^ and D23580 is an invasive epidemic strain originally isolated from a child in Malawi.^24^

We first showed that neither of the strains caused an increase in light transmission in washed platelets demonstrating that they are unable to directly induce platelet aggregation (Supplementary Figure S1). In line with results in Arman *et al*,^18^ the addition of fibrinogen did not lead to aggregation (data not shown). Addition of pooled human IgG with or without fibrinogen also did not lead to aggregation (data not shown), consistent with the results obtained by Arman *et al*. for *S. pneumoniae*.^18^

**Figure 1:**
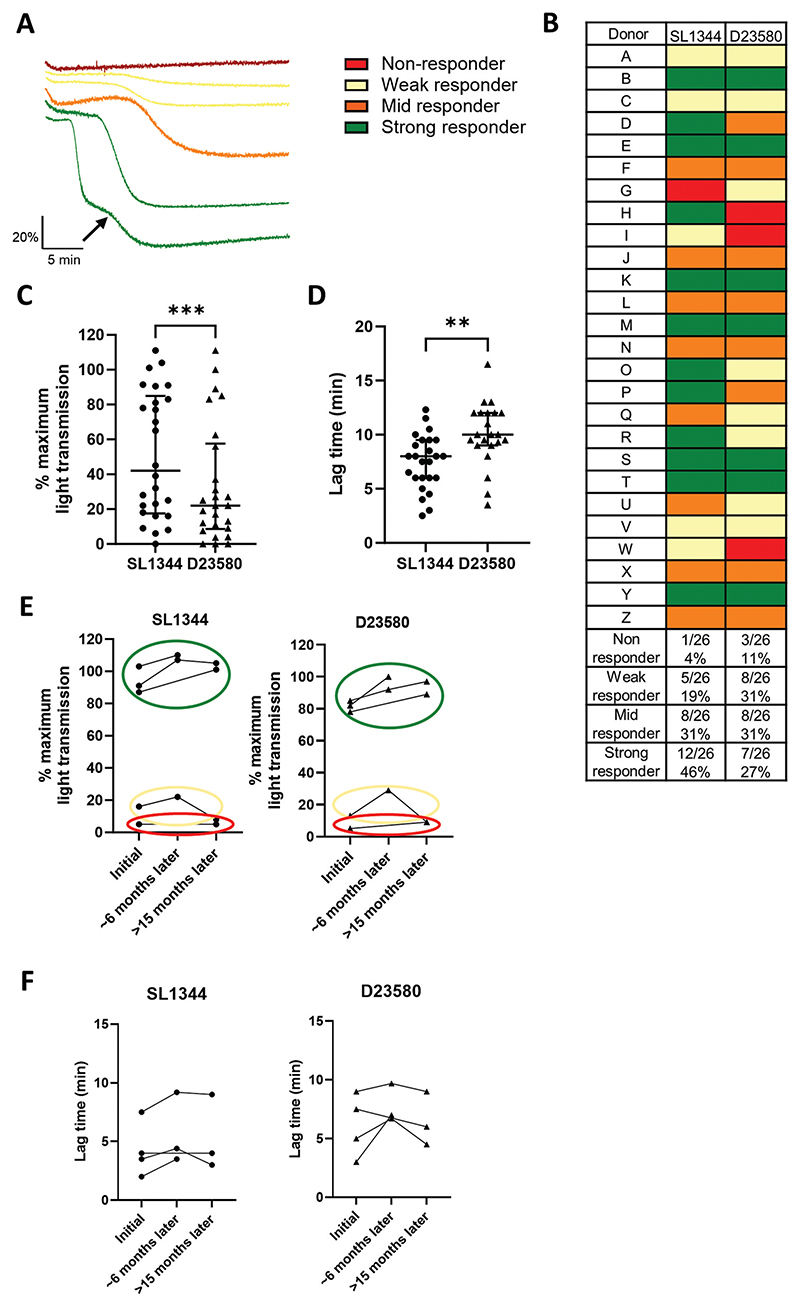
*Salmonella* activates platelets in a donor and strain dependent manner in platelet rich plasma (PRP). PRP was stimulated with a final concentration of ~3.3 x 10^8^ CFU/ml STm SL1344 or STm D23580. Colour coding: strong responders (green; >60% maximum aggregation), mid responders (orange; 20-60% maximum aggregation), weak responders (cream, <20% maximum aggregation, but still with a curve), and nonresponders (red, no aggregation). (A) Representative traces from the different colourcoded categories to STm SL1344. The arrow shows the point at which the secondary wave of aggregation begins, demonstrating the biphasic response in this donor. (B) Table summarising responses to SL1344 and D23580 from n = 26 healthy donors. (C) Numerical maximum light transmission results for responses to SL1344 and D23580. n =26. Bars are median and inter-quartile range. Wilcoxon test, *** p ≤0.001. (D) Lag times taken for aggregation to occur to (n = 25) and D23580 (n = 22). Bars are median and inter-quartile range. Unpaired t-test ** p ≤ 0.01. (E) Maximum light transmission percentages of donors over time n = 5. ~6 month time point runs from 182-196 days after initial measurement, >15 months covers tests >450 days after initial measurement (F) Lag times of donors over time. n = 4.

Many bacterial species can indirectly activate platelets through bridging proteins such as IgG, fibrinogen or complement.^25^ To establish whether this was the case for STm, we monitored activation in platelet-rich plasma (PRP) using light transmission aggregometry. Both strains were able to cause platelet aggregation, but with marked differences between individual donors, as well as between the STm strains themselves (Figure 1). In all cases, the increase in light transmission was blocked in the presence of the αIIbβ3 blocker eptifibatide, demonstrating that responses were true aggregation and not agglutination (Supplementary Figure S2). To ensure we were using the optimal concentration of bacteria, we initially tested final concentrations from 1 × 10^7^ CFU/ml to 3.3 × 10^8^ CFU/ml. No responses were seen at 1 × 10^7^ CFU/ml, but similar aggregation responses were observed at 1 × 10^8^ CFU/ml and 3.3 × 10^8^ CFU/ml for nearly all donors. Therefore, the bacterial concentration of 3.3 × 10^8^ CFU/ml was chosen so we could rule out bacterial concentration as a factor in the variation in responses

To characterize the variation in further detail, we categorized the donors into different response groups (Figure 1A,B), with example aggregation curves for SL1344 shown in Figure 1A. Strong responders, shown in green, had a maximum platelet aggregation that was similar in magnitude to that of classical platelet agonists such as ADP. The two examples of strong responders shown in Figure 1A differed in lag time and pattern of response, with a biphasic curve observed in one donor. Mid responders are shown in orange and had maximum aggregation values between 20% and 60%. Weak responders had a maximal aggregation value of <20%, with two examples shown in cream (one with a more obvious curve than the other). Non-responders showed no response to the bacteria at all (despite normal responses to the platelet agonist, TRAP-6, data not shown), with an example trace shown in red. Figure 1B shows that while some donor responses are in the same category for both strains (e.g. Donors A and B), this was not the case for all donors (e.g. Donors D, G and H). In a total of 26 donors (age range 20–64 years; 11 female, 15 male), the median maximum aggregation response to SL1344 was 42% with an inter-quartile range (IQR) of 17.5–84.9% (Figure 1C). Bacterial-induced platelet aggregations have a characteristic lag time before platelet aggregation occurs, unlike traditional agonists,^26^ and this was observed in aggregations to STm. The lag times for onset of aggregation ranged from 2.5 to 12.5 min (median 8 min, IQR 6–9.5 min; Figure 1D). Responses tended to be weaker to D23580 than to SL1344, with a median aggregation response of 22% (IQR 8.6–57.6%) (Figure 1C) and a longer lag time median of 10 min (IQR 9–12 min) (Figure 1D). There was no clear link between sex or age and whether a donor was a responder or non-responder.

With such variation between donors, it was important to assess whether donor responses remained stable over time or not. The responses of donors to the two STm stimuli were tested at 6 and/or 15 months after the first test. As shown in Figure 1E, platelet aggregation responses remained in the same response category across both strains. Lag times also remained broadly similar (Figure 1F).

### *Salmonella*-induced platelet aggregation is mediated by FcγRIIA and secondary mediators

**Figure 2:**
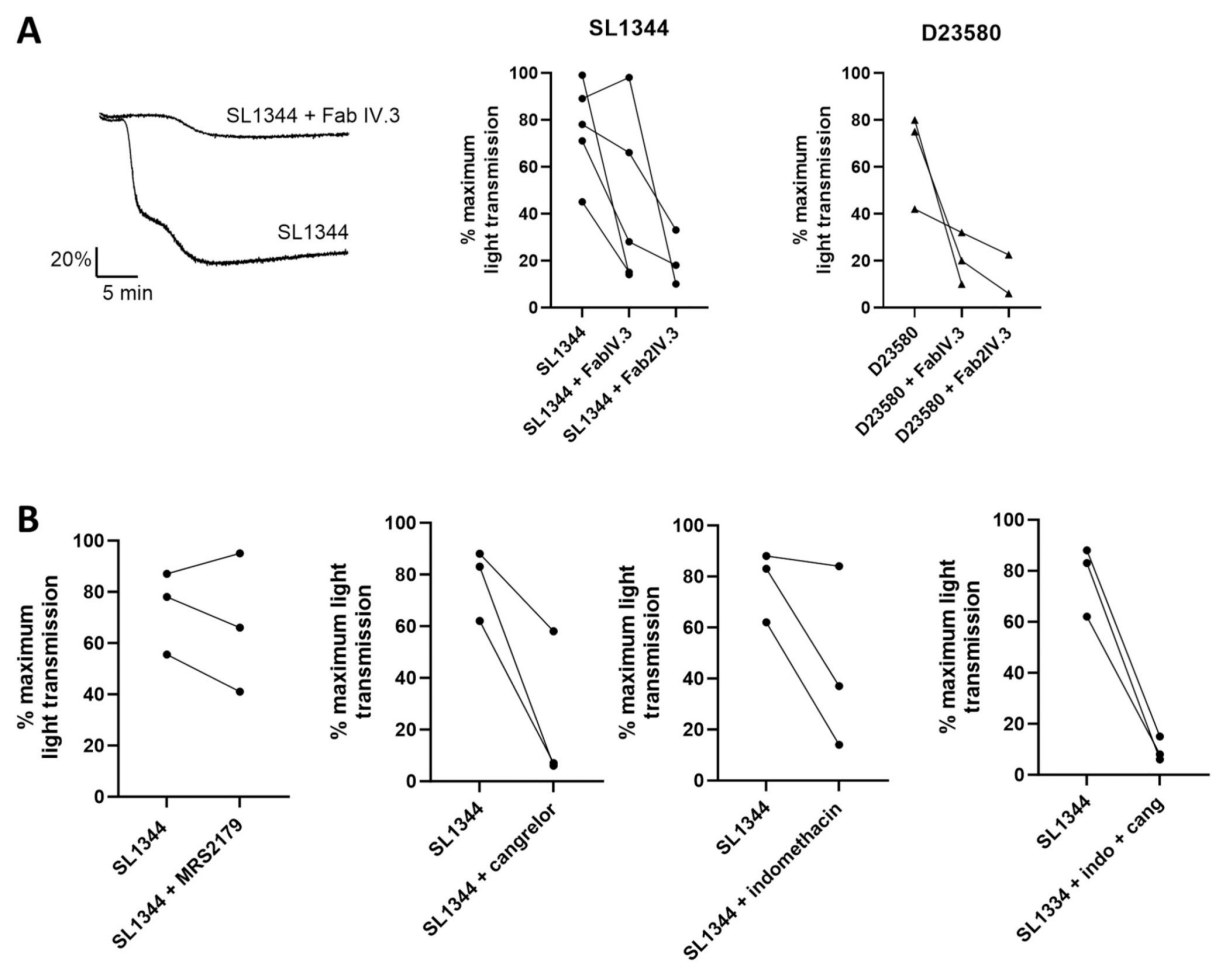
*Salmonella*–induced platelet aggregation is mediated by FCγRIIA and secondary mediators. (A) PRP was pre-incubated with 20 μM F(ab) IV.3 or 10 μM F(ab)2 IV.3 or vehicle for 3 min before stimulation with a final concentration of ~3.3x10^8^ CFU/ml of STm SL1344 or D23580. n ≥3 (B) PRP was pre-incubated with either 20 μMMRS2179, 1 μM cangrelor, 10 μM indomethacin, vehicle control or both indomethacin and cangrelor for 3 min. STm SL1344 was then added at a final concentration of ~3.3x10^8^ CFU/ml. n = 3.

We next set out to characterize the STm-platelet interactions. The receptor FcγRIIA and its ligand IgG have been implicated in many bacterial-platelet interactions.^18,27,28^ We therefore tested whether FcγRIIA was required for STm-induced platelet aggregation. The use of the F(ab) fragment of FcγRIIA blocking monoclonal antibody IV.3 reduced almost all aggregation responses to basal levels (Figure 2A). Where this was not achieved, use of the higher affinity F(ab)_2_ fragment was used, which caused full inhibition.

As shown in Figure 1A, in some donors the aggregation response is biphasic, suggesting the need for feedback agonists. To investigate this, we assessed the effect of inhibitors of the two major feedback agonists, ADP and thromboxane A_2_, in strongly responding individuals (Figure 2B). Inhibiting signaling through the ADP receptor P2Y_1_ with MRS2179 had no significant effect on the time course of the response or the level of aggregation. In contrast, addition of the P2Y_12_ inhibitor cangrelor blocked aggregation in two out of three donors and reduced the response in the remaining donor. The cyclooxygenase inhibitor indomethacin had a similar effect to that of cangrelor, with the magnitude of response unchanged in one donor but with a slight increase in lag time. When both cangrelor and indomethacin were used in combination, platelet aggregation was abolished in all three donors, confirming the requirement for secondary mediators (Figure 2B).

### A plasma component is responsible for donor variation in platelet aggregation responses to *Salmonella*

Our next goal was to identify what was behind the variation in donor platelet responses to STm. As the PRP used was not normalized to a standard platelet count (as recommended in ISTH guidelines^29,30^), we first checked that differing platelet counts in PRP were not accounting for the inter-donor variability. Platelet counts obtained at the time of testing were plotted against maximum light transmission levels and this showed there was no correlation between platelet count and levels of aggregation (Suplementary Figure S3).

To establish whether the donor variation in platelet aggregation to STm was due to donor differences in platelets, plasma or both, “donor swap” assays were carried out. ADP-sensitive washed platelets and platelet poor plasma (PPP) were obtained from strong and weak responders. As controls, the strong responders’ platelets were resuspended in the strong responders’ plasma, and the same was carried out with the weak responder. To test whether the platelet or plasma component had a stronger influence over the donor variability, the strong responders’ platelets were resuspended in the weak responders’ plasma, and vice versa.

**Figure 3:**
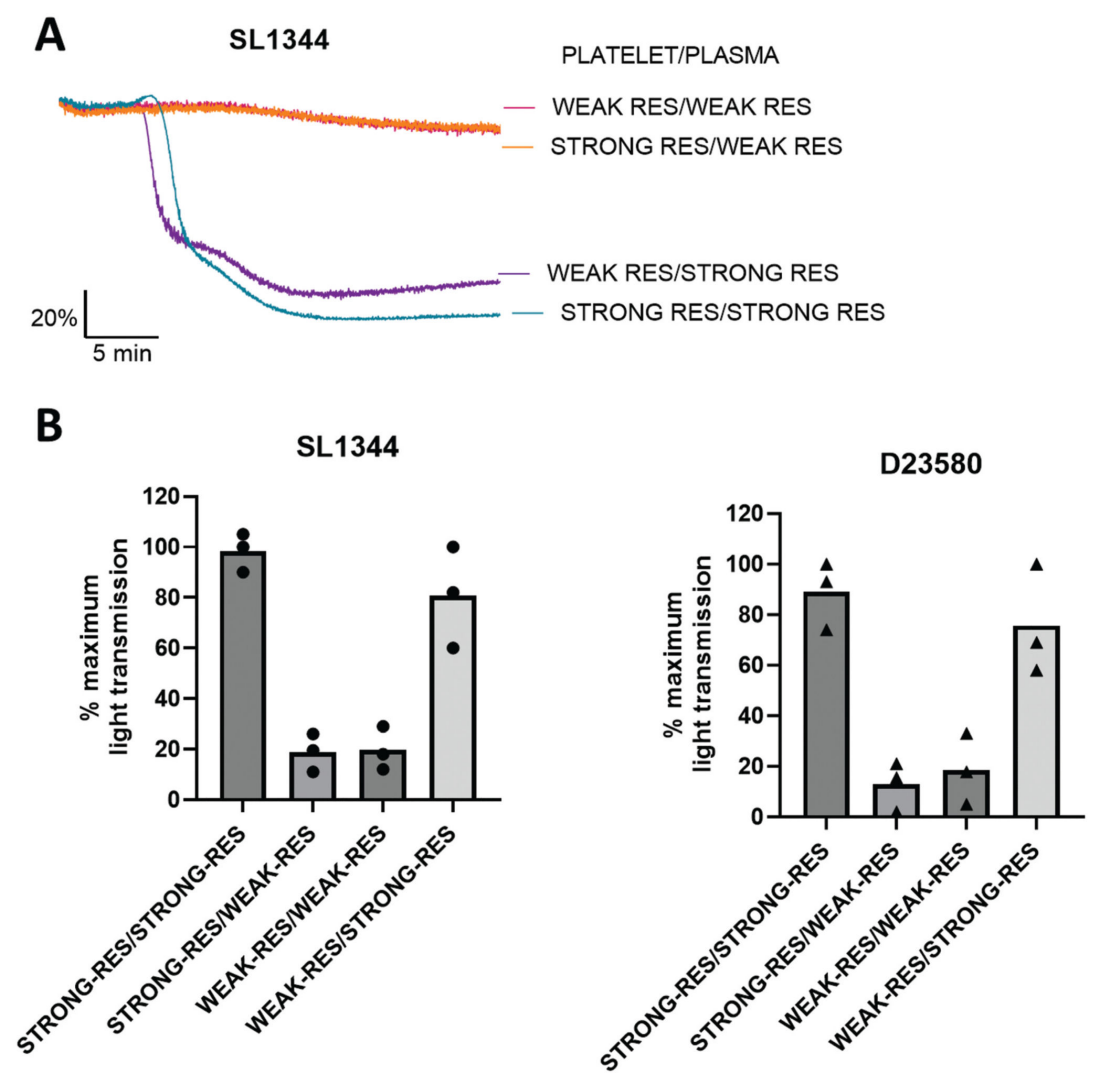
A plasma component is responsible for donor variation in platelet aggregation responses to *Salmonella*. Two washed platelet pellets from a strong and weak responder were obtained using an ADP-sensitive preparation and resuspended in ACD-free PPP from both donors. Platelet concentrations were 2.3-2.5 x 10^8^/ml depending on PPP amounts obtained for dilution. The final concentration of bacteria added was ~3.3x10^8^ CFU/ml. (A) Representative traces of a donor swap experiment using SL1344. (B) Labels show platelet donor first, followed by plasma donor for SL1344 and D23580, bars represent mean. n =3.

As shown in Figures 3A,B, the combination of the strong responders’ platelets with the weak responders’ plasma caused aggregation levels equivalent to that of the weak responder control. In contrast, the weak responders’ platelets resuspended in the strong responders’ plasma gave equivalent aggregation responses to the strong responder control. Together, these results suggest that a plasma component is responsible for the inter-donor variation in response to STm.

### Anti-*Salmonella* antibodies are required for aggregation, and their levels correlate with the strength of the platelet response

**Figure 4:**
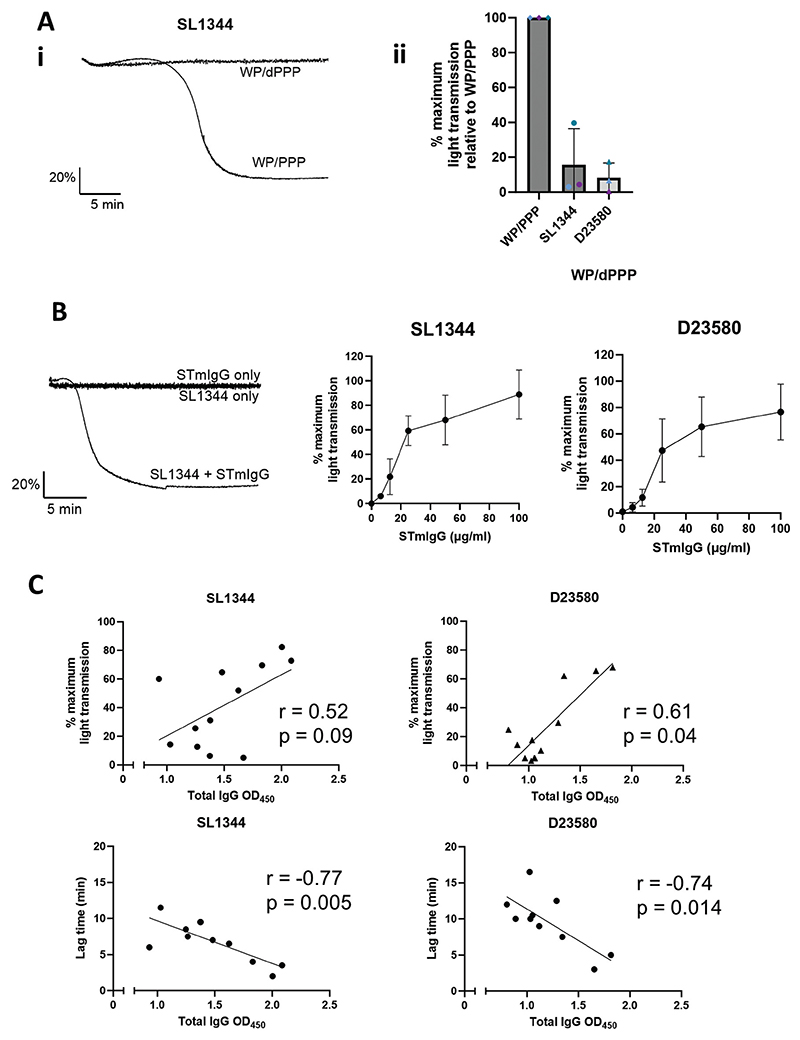
*Salmonella -*binding antibodies are required for aggregation and their levels correlate with the strength of platelet response. (A) ADP sensitive washed platelets were resuspended in either normal plasma (WP/PPP) or *Salmonella*-binding antibody depleted plasma (WP/dPPP). Bacteria was added to a final concentration of ~3.3x10^8^ CFU/ml. Ai) Representative traces from a strong responder. Aii) Pooled data from n = 3. Colours represent separate donors – blue and purple were strong responders, teal a weak responder. Data are presented as WP/dPPP responses to the three bacterial relative to the control WP/PPP level taken as 100% maximum aggregation. (B) Dose response curves to the *Salmonella* strains using an ADP sensitive washed platelet preparation with the addition of 100 μg/ml rabbit anti-*Salmonella* IgG (STmIgG). n = 3. Bars are mean ± SD. (C) Total IgG levels to both strains were measured using an in-house ELISA, and results correlated against maximum light transmission levels (top 2 graphs, n = 12) and lag time (bottom 2 graphs, n ≥ 10) from Fig 1. Spearman rank correlation coefficient was calculated for the non-parametric maximum light transmission results. Pearsons correlation coefficient was calculated for the parametric lag times.

With the focus on identifying a plasma component responsible for donor variation in responses, and the knowledge that inhibiting FcγRIIA abolished aggregation responses, we examined the requirement of anti-STm IgG antibodies for platelet aggregation. Using a similar method to the donor swap assay above, we took PPP from donors and depleted it of anti-STm antibodies. We then resuspended the ADP sensitive washed platelet pellets in either their normal PPP (WP/PPP), or the anti-STm antibody depleted plasma (WP/dPPP). As shown in Figure 4A, the washed platelets resuspended in normal plasma aggregated as expected, but depleting the anti-STm antibodies from plasma abolished aggregation. Consistent with this, we show that the addition of a rabbit anti-*Salmonella* polyclonal antibody (STmIgG) to washed platelets before stimulating with STm-induced aggregation in a dose-dependent manner (Figure 4B).

The levels of total IgG binding to whole STm bacteria by our donor serum were then tested by ELISA and levels correlated against the maximum platelet aggregation level and lag time. This revealed higher anti-STm IgG levels correlated with higher platelet aggregation levels, and faster lag times (Figure 4C).

**Figure 5:**
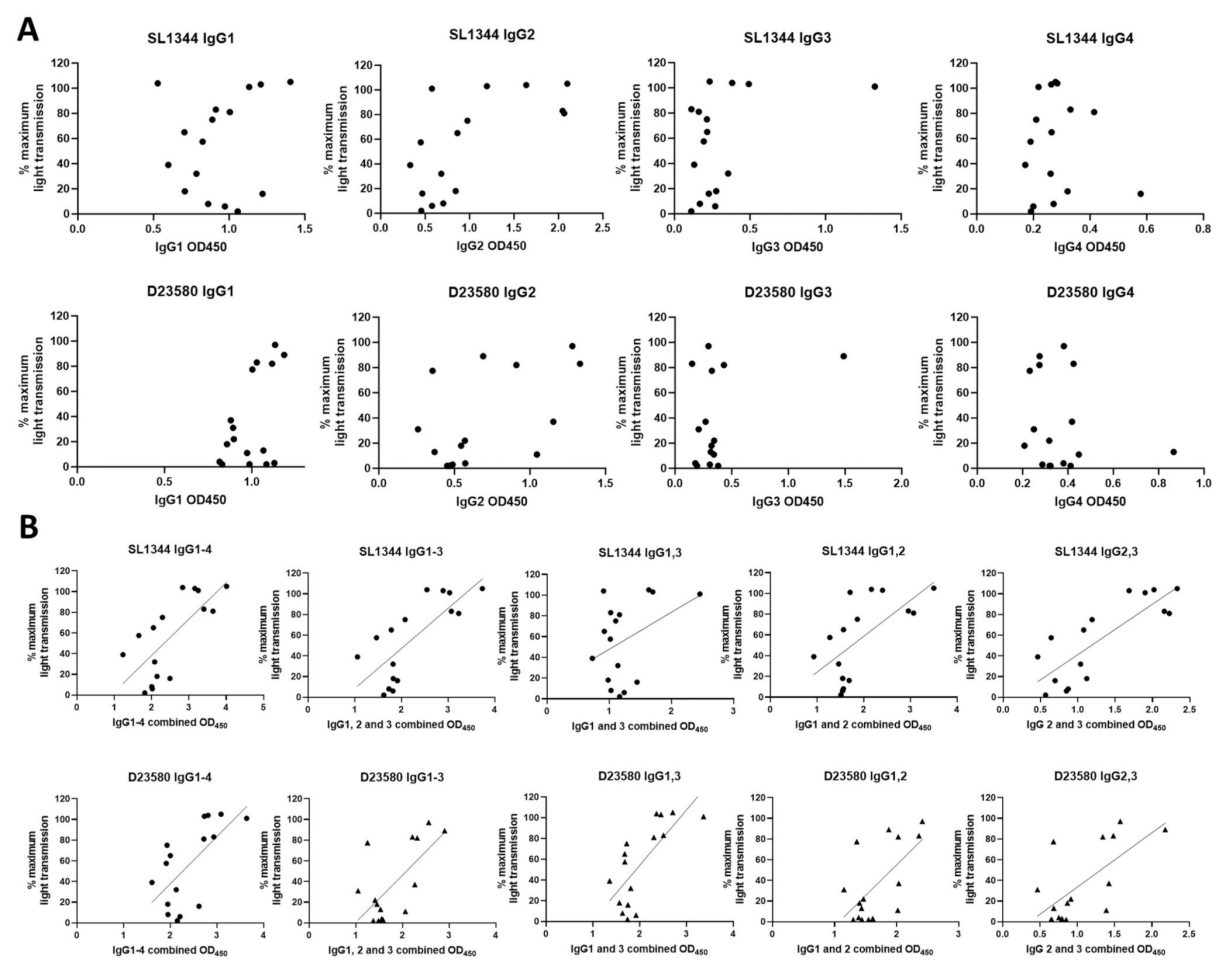
Individual *Salmonella*-binding IgG subclass levels do not correlate with platelet aggregation responses to *Salmonella*, but combining subclasses reveals a correlation. In house ELISAs were carried out to measure levels of anti-*Salmonella* IgG subclass antibodies to each of the three strains. Results were correlated against maximum aggregation. ODs were combined from multiple subclasses, and their totals correlated against maximum aggregation in the bottom half of the figure. n = 16. Spearman correlations for these graphs are in Supp Fig 4. Lines are simple linear regression.

To further investigate whether a specific IgG subclass was linked to platelet aggregation, whole bacteria ELISAs were carried out to assess levels of anti-STm IgG1, 2, 3 and 4. As seen in Figure 5A and Supplementary Figure S4A, none of the individual subclasses show clear correlation patterns. However, when combining subclasses, correlations were found, suggesting that the presence and total amounts of antibody are more important for platelet aggregation than the presence of a specific IgG subclass (Figure 5B and Supplementary Figure S4B).

## Discussion

We show that STm can activate platelets from healthy donors in a donor- and strain-dependent manner. Binding of STm by antibody is needed to activate platelets via FcγRIIA, with higher levels of antibody leading to stronger platelet aggregation and shorter lag times.

Traditionally, bacterial-induced platelet aggregations have been viewed as all-or-nothing responses, with either full aggregation or no aggregation observed.^25^ The STm-induced platelet aggregation responses observed here challenge this concept, with differing levels of activation being dependent on the donor, and the aggregation levels varying in washed platelets according to the concentration of anti-*Salmonella* antibody added. While studies on various Gram-positive strains have revealed donor variations in lag time,^31,32^ the only study finding variation in maximum aggregation responses looked at platelet responses to heat aggregated human IgG.^33^ Variation between strains however is a more common finding, being shown in bacteria such as *Cutibacterium acnes, Streptococcus oralis* and *Helicobacter pylori*.^28,34,35^

Physiologically, the average blood culture result of iNTS patients was shown in one study to be just 1 CFU/ml.^4^ This is several magnitudes lower than the amount used in this study. However, it is not uncommon for bacterial-platelet interaction studies to use high concentrations of bacteria. The concentration of *Salmonella* used in this study is in line with other bacterial-platelet interaction studies, including Arman *et al*. (3 × 10^8^ CFU/ml, S gordonii), Corcoran *et al*. (4 × 10^8^ CFU/ml H pylori) and Kerrigan *et al*. (7 × 10^9^ CFU/ml, S sanguinis). The differences in the levels of bacteria required likely reflect the timescales involved in the aggregation events. In our assays, aggregation takes place within 30 min. While the duration of illness in humans with iNTS has limited study data,^36^ in pre-clinical models it can be in the order of weeks. In a mouse model of *Salmonella* infection, thrombi occur in the spleen and liver after 1 and 7 days respectively.^37,38^ The high concentrations of bacteria used in this assay allow a fast response, providing a model in which to elucidate the potential mechanisms involved at the molecular level.

Although we showed correlations between total anti-STm IgG levels, no role for individual subclasses was revealed. Instead, the overall combination of IgG antibody subclasses binding to STm allows aggregation proportional to the levels present. Nevertheless, FcγRIIA has different affinities for the different subclasses (IgG3>IgG1>IgG4>IgG2^39^), and these affinities are further influenced by the single nucleotide polymorphism (SNP) at position 131. For example, if an individual is homozygous for histidine then FcγRIIA has an increased affinity to IgG1 and IgG2, compared to when they are homozygous for arginine at this position.^40,41^ These affinity differences could potentially be enough to alter the amount of antibody bound by a platelet at any one time, influencing whether a strong or weak response is provoked. Despite the donor swap assays ruling out the platelets as a cause of donor variation, the donors used in the assay were the stronger and weaker responders, so the high levels of antibody present in the stronger responders could have compensated for any differences in platelet responses. We were unable to test mid-responders in this assay due to a reduction in platelet aggregation observed upon resuspending platelet pellets in PPP. This is similar to the phenomenon reported by Cattaneo et al.,^30^ where dilution of PRP with PPP to normalize platelet count inhibited the platelet aggregation responses. To circumvent this, we optimized the platelet preparation by adding apyrase, which helped limit the reduction in platelet sensitivity upon dilution in PPP, but still not to the extent whereby mid-responders were able to respond in their own PPP to a level close to their PRP response, limiting our experiments to strong/weak responders only. Therefore, further work would be needed to see whether FcγRIIA levels on the platelet surface and their polymorphisms could also be playing a role in donor variation.

As well as FcγRIIA, other platelet receptors could be playing a role in *Salmonella-*induced platelet aggregations. In the mouse model of *Salmonella* infection, platelet CLEC-2 was identified as being vital to the thrombotic response in the liver.^38^ However, mouse platelets have over 10× the amount of CLEC-2 on their platelets as humans,^42,43^ and do not express FcγRIIA. This highlights the importance of human studies alongside mouse studies, and further work would be required to assess whether CLEC-2 is involved in the mechanism in human platelets. Furthermore, human platelets also express complement receptors, such as gC1q/p33 and cC1qR. Complement protein C1q can bind antibody-bacteria complexes, which could provide a further mechanism of bacterial-recognition and activation of the platelet. Complement has been implicated in platelet aggregation responses to S *sanguinis*, with removal of complement abolishing the aggregation responses seen.^32^ Again, work beyond the scope of this study would need to be carried out to assess this.

Another area that requires further exploration is the source and antigen-specificity of the anti-STm antibodies involved in this process. Nearly all adults have anti-STm IgG antibodies.^44,45^ The O-antigen component of LPS is an immunodominant antigen and major target of antibodies against whole STm bacteria, reflecting the sheer prevalence of this antigen on the bacterial surface.^46^ Since depletion of anti-STm whole bacteria antibodies, which efficiently removes anti-LPS O-antigen antibodies,^47^ results in loss of platelet aggregation it suggests that anti-LPS antibodies induced to STm are important for FcγRIIA activation. However, antibodies induced to the O-antigen in other bacterial pathogens can potentially cross-react with STm^44,48,49^ and these may also contribute. As aggregation responses to STm remained constant over the sampling period, implying antibody levels are remaining constant, it may be that a combination of STm-specific and cross-reactive antibodies are present and can contribute to platelet activation.

Differences in platelet aggregation occurred between the two STm strains used here, with lower average maximum aggregation and longer average lag times for D23580 than SL1344. Although in many ways these strains are highly similar, hence both being STm, there are differences between the two and they are from different sequence type groups. The invasive disease-associated D23580 strain was first isolated from the bloodstream of a Malawian child, whereas SL1344 is of bovine origin and is widely used in laboratories around the world. D23580 shows some evidence of host adaptation to humans with some genome degradation relative to SL1344. Two of the genes degraded in comparison to SL1344 are putative outer or integral membrane proteins, and one deletion of a secreted protein has also been noted.^24^ Whether or how these could influence platelet aggregation responses directly is unclear.

The observation that higher amounts of anti-STm antibody cause higher levels of platelet aggregation has implications for the immunothrombotic or thromboinflammatory responses to STm infection. In someone who already has high amounts of anti-STm antibody present at the time of pathogen encounter then antibodies can bind the bacteria leading to platelet activation and thrombus formation around the bacteria. This immunothrombotic response to a minimal number of bacteria could help stop further replication and dissemination of the bacteria throughout the bloodstream. This entrapping of *Salmonella* in human platelet aggregates *in vitro* has previously been shown by Beristain-Covarrubias et al.^37^ In this instance, having a high level of anti-STm antibodies is likely to be helpful. On the other hand, in those who have low or no anti-STm antibodies at the time of infection, higher levels of antibody would not be present until later, when infection is established. By this point, there is the potential for bacterial numbers to be at high levels. Here, high levels of antibody plus high levels of bacteria could be providing more opportunities for platelet activation and thrombus formation, with the potential of leading to a dysregulated thromboinflammatory response, worsening disease.

To conclude, we show that STm can activate human platelets in the presence of anti-STm antibodies. Variation in donor responses to STm is linked to the levels of anti-STm antibody present. These findings begin to reveal the factors behind differences in individual thromboinflammatory responses to infectious agents, unveiling potential avenues for therapeutic intervention.

## Supplementary Material

Supplementary Figures 1-2

Supplementary Figures 3-4

## Plain Language Summary

- *Salmonella* is widely known as a bacterial pathogen that causes diarrhea, with recovery occurring after about a week. However, there exists another, less well known and more severe form of *Salmonella*, called invasive non-typhoidal *Salmonella*. This type of *Salmonella* infection gets across the gut barrier, enters the bloodstream and spreads through the body. There are over half a million of these kinds of invasive *Salmonella* infections globally every year, leading to over 75 000 deaths. The majority of these are in sub-Saharan Africa but also are significant causes of harm in the elderly or those with compromised immune systems. When the bacteria are in the bloodstream, they can come into contact with various different types of cells, including platelets, the cells that cause blood to clot. Sometimes the presence of bacteria in the blood can cause platelets to clump together, leading to blood clots, which can block the blood vessels and cause organ damage, and death. The ability of *Salmonella* bacteria to cause clots has not been investigated in depth.

### What is new?

- We show that the bacteria that cause invasive *Salmonella* infections are able to trigger platelets to clump together and form a blood clot. However, the response of platelets in blood plasma from donors varies widely - some people’s platelets do not clump together much, whilst others form large clots. We show that the amount people’s platelets clot is linked to the level of antibodies that bind to *Salmonella*.

### What is the impact?

- There are limited treatment options for invasive non-typhoidal *Salmonella* infections due to increasing antimicrobial resistance. Therefore, understanding the mechanisms behind how *Salmonella* interacts with blood cells such as platelets could help direct further research to identify new treatment options for these infections.
