# Supplementary figures and images for: Platelet aggregation responses to Salmonella Typhimurium are determined by host anti-Salmonella antibody levels
Source: Platelets. Author manuscript; Available in PMC 2025 May 15. (PMC7617673; doi:10.1080/09537104.2024.2437241)

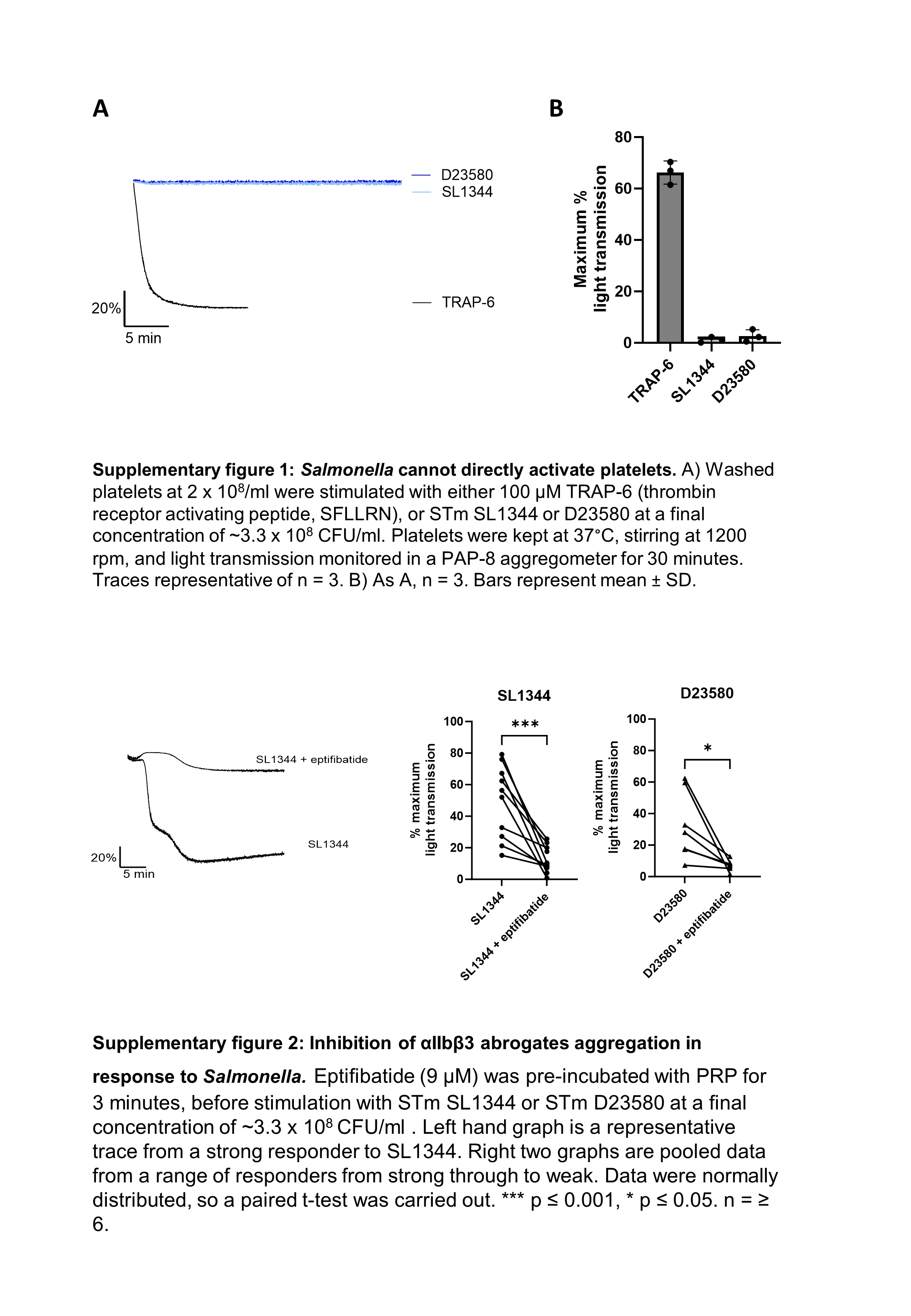

Supplement: Supplementary Figures 1-2 [file EMS205385-supplement-Supplementary_Figures_1_2.TIF]

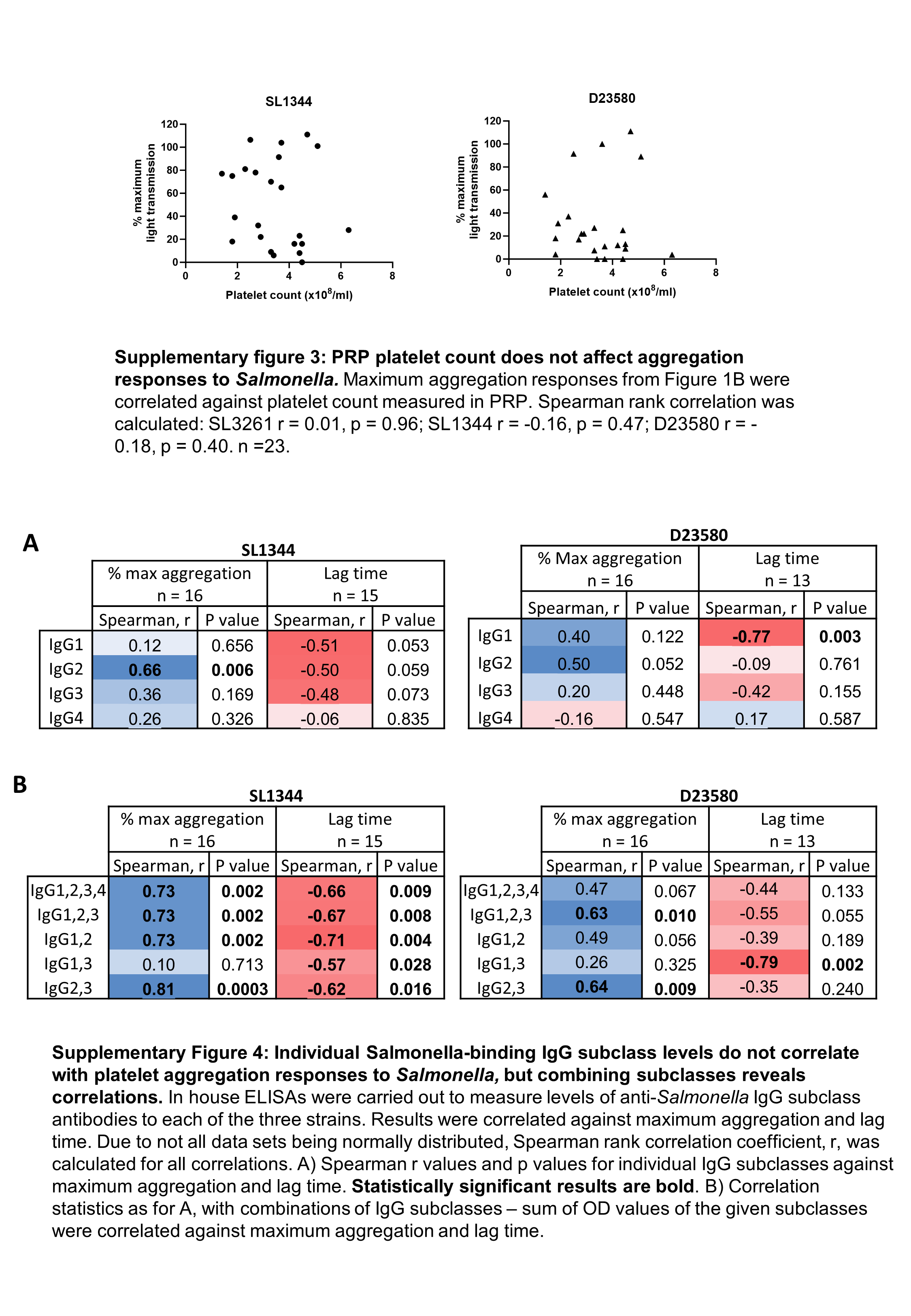

Supplement: Supplementary Figures 3-4 [file EMS205385-supplement-Supplementary_Figures_3_4_.TIF]
